# Aldose Reductase Inhibition Prevents Metaplasia of Airway Epithelial Cells

**DOI:** 10.1371/journal.pone.0014440

**Published:** 2010-12-28

**Authors:** Umesh C. S. Yadav, Leopoldo Aguilera-Aguirre, Kota V. Ramana, Istvan Boldogh, Satish K. Srivastava

**Affiliations:** 1 Department of Biochemistry and Molecular Biology, University of Texas Medical Branch, Galveston, Texas, United States of America; 2 Department of Microbiology and Immunology and Sealy Center for Molecular Medicine, University of Texas Medical Branch, Galveston, Texas, United States of America; Comprehensive Pneumology Center, Germany

## Abstract

**Background:**

Goblet cell metaplasia that causes mucus hypersecretion and obstruction in the airway lumen could be life threatening in asthma and chronic obstructive pulmonary disease patients. Inflammatory cytokines such as IL-13 mediate the transformation of airway ciliary epithelial cells to mucin-secreting goblet cells in acute as well as chronic airway inflammatory diseases. However, no effective and specific pharmacologic treatment is currently available. Here, we investigated the mechanisms by which aldose reductase (AR) regulates the mucus cell metaplasia *in vitro* and *in vivo*.

**Methodology/Findings:**

Metaplasia in primary human small airway epithelial cells (SAEC) was induced by a Th2 cytokine, IL-13, without or with AR inhibitor, fidarestat. After 48 h of incubation with IL-13 a large number of SAEC were transformed into goblet cells as determined by periodic acid-schiff (PAS)-staining and immunohistochemistry using antibodies against Mucin5AC. Further, IL-13 significantly increased the expression of Mucin5AC at mRNA and protein levels. These changes were significantly prevented by treatment of the SAEC with AR inhibitor. AR inhibition also decreased IL-13-induced expression of Muc5AC, Muc5B, and SPDEF, and phosphorylation of JAK-1, ERK1/2 and STAT-6. In a mouse model of ragweed pollen extract (RWE)-induced allergic asthma treatment with fidarestat prevented the expression of IL-13, phosphorylation of STAT-6 and transformation of epithelial cells to goblet cells in the lung. Additionally, while the AR-null mice were resistant, wild-type mice showed goblet cell metaplasia after challenge with RWE.

**Conclusions:**

The results show that exposure of SAEC to IL-13 caused goblet cell metaplasia, which was significantly prevented by AR inhibition. Administration of fidarestat to mice prevented RWE-induced goblet cell metaplasia and AR null mice were largely resistant to allergen induced changes in the lung. Thus our results indicate that AR inhibitors such as fidarestat could be developed as therapeutic agents to prevent goblet cell metaplasia in asthma and related pathologies.

## Introduction

In sensitized individuals, exposure to environmental allergens (such as pollens, molds, dust mites) induce inflammatory response in the airway characterized by inappropriate Th2 response [1], [2], such as secretion of cytokines, especially Th2 type [3], [4]. The altered cytokine levels and subsequent changes in airway redox status lead to goblet cell metaplasia, transforming airway epithelial cells into mucus secreting cells, which release overwhelming amounts of mucin, especially mucin5AC, which obstructs the smaller alveoli and causes obstruction in breathing in asthma [5]–[10]. Excessive mucus secretion often becomes life threatening [11]. Since oxidative stress exacerbates pathological changes in the airway including goblet cells metaplasia and hyperplasia leading to mucin hypersecretion [12]–[14], blocking the oxidative stress-induced signaling should be effective in decreasing mucin production and airway obstruction in asthma.

Th2 cytokines, especially IL-4 and IL-13, induce the phosphorylation of STAT (signal transducer and activator of transcription) -6 which transcribes genes involved in metaplasia leading to excessive mucus production [5], [15]–[19]. Recently, SPDEF (SAM-pointed domain–containing ETS transcription factor), a member of the Ets family of transcription factors, has been implicated in goblet cell metaplasia in response to allergen or IL-13 challenge [20]. Park et al (2007) have indeed, demonstrated increased goblet cell differentiation in SPDEF transgenic mice in the respiratory epithelium [21]. Studies have also demonstrated the role of STAT-6 and SPDEF in airway inflammation and resultant mucin production [22]–[25]. Since cytokines and growth factors are known to generate ROS which act as secondary messenger, and AR has been implicated in ROS-mediated inflammation resulting in the lung pathology [26], [27]. Our hypothesis is that inhibition of AR should ameliorate the ROS-induced inflammatory signals in the airway and thereby decrease/block the inflammation and resultant pathological outcomes.

**Figure 1:**
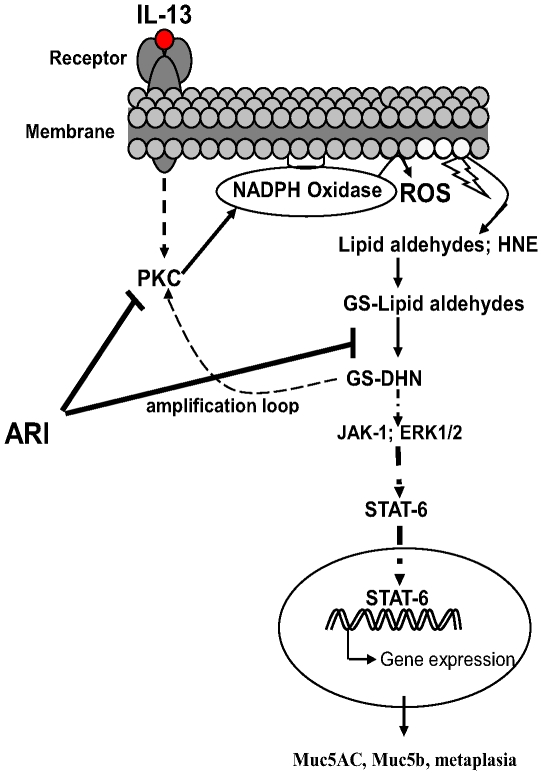
Prevention of redox signaling by AR inhibition. Stimulation with Ragweed pollens, allergens, growth factors, or cytokines cause increased ROS production inside the cells. The ROS could oxidize membrane lipids to form lipid aldehydes such as 4-hydroxynonenal (HNE), which readily reacts with glutathione and form GS-conjugates such as GS-HNE. AR catalyzes the reduction of GS-HNE into GS-DHN. The latter is known to activate various downstream protein kinases such as PKC, PI3K and MAPK activating transcription factors NF-κB and AP-1 which transcribe various inflammatory markers, including IL-13 which in paracrine fashion induces more ROS formation. The GS-DHN could also phosphorylate JAK-1 and MAPK such as ERK1/2, eventually phosphorylating and activating STAT-6 which translocate to the nucleus and transcribe genes including mucin genes leading to goblet cell metaplasia. The continuously formed GSDHN may amplify the signaling loop by increased ROS production via PKC/NOX pathway. Inhibition of AR blocks the initial as well as the amplification loop and prevents ROS-induced pathological changes [Ref. 31], [32].

We have recently demonstrated that sensitization and challenge of mice with RWE resulted in increased expression of Th2 cytokines such as IL-4, IL-5 and IL-13 in the lung. Also a large amount of Mucin 5AC was detected in the bronchoalveolar lavage. Inhibition of AR significantly decreased these changes indicating that AR could be crucial in the allergen-induced airway inflammation [26]. However the exact role of AR in goblet cell metaplasia in allergic asthma is not clear. We have previously shown that AR catalyzes the reduction of lipid-aldehydes and their glutathione conjugates, formed inside the cells during oxidative stress. The reduced GS-lipid aldehyde conjugates activate upstream kinases resulting in the activation of transcription factors and subsequent expression of inflammatory genes [28]–[32]; [Fig. 1]. Recently, Mandal et al (2010) demonstrated that IL-13 stimulates ROS synthesis in intestinal epithelial cells which in turn positively regulates phosphorylation of ERK1/2 and STAT6 and also stimulates expression of NADPH oxidase [33]. Studies have demonstrated that oxidative stress induces AR which in turn mediates JAK-STAT signaling in the pathophysiology of myocardial ischemic injury [34], [35]. Since IL-13-induced STAT-6 activation is implicated in the goblet cell metaplasia, we hypothesized that allergen-induced IL-13 production in allergic asthma would result in the exacerbated oxidative stress that would cause increase in AR in the airway cells leading to altered cellular signaling that induce transformation of epithelial cells into goblet cells. Therefore, we investigated the role of AR in Th2 cytokine, IL-13 -mediated activation of STAT-6 signaling that is known to cause goblet cell metaplasia and enhanced mucus production in the airway [19], [23]. We used cultured primary airway epithelial cells grown on the air-liquid interface (ALI) and RWE-induced murine model of allergic asthma. To delineate the role of AR in metaplasia of airway epithelial cells we used a specific inhibitor of AR, fidarestat which has already undergone 52 weeks of phase III clinical trial for diabetic neuropathy and was found to have no irreversible side effects. Our results demonstrated that AR inhibition significantly prevented development of goblet cell metaplasia induced by IL-13 in human airway epithelial cells and by allergen (RWE) in mice.

## Results

### AR inhibition prevents IL-13 –induced ROS levels in SAEC

**Figure 2:**
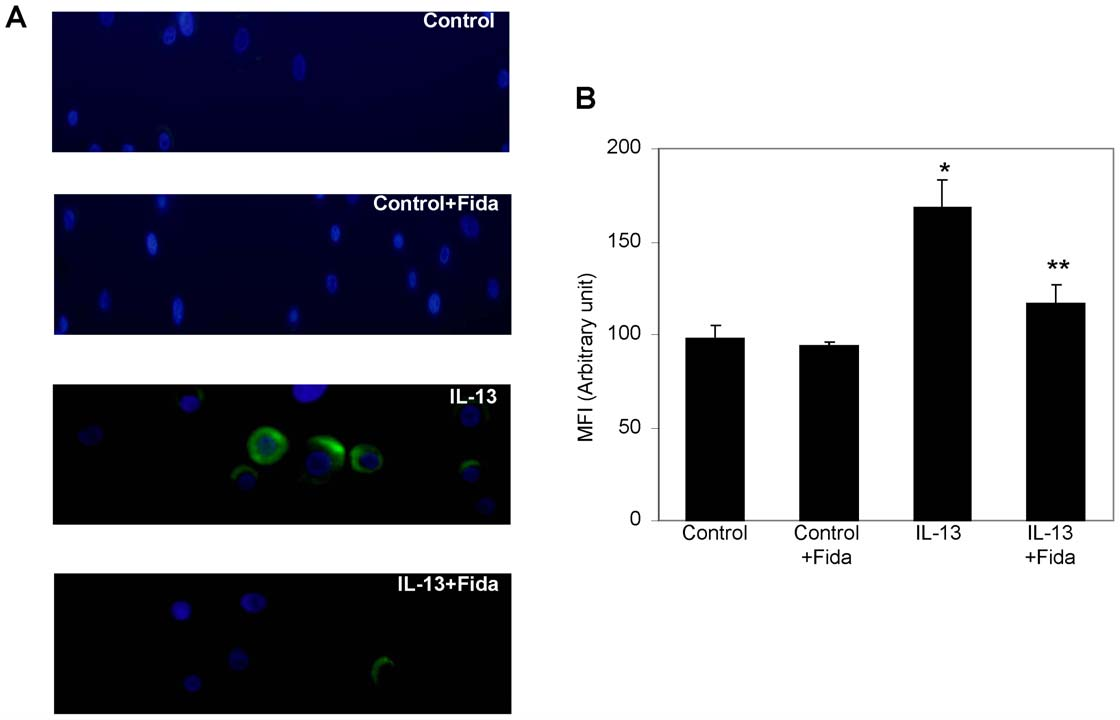
Inhibition of AR prevents IL-13-induced ROS production in SAEC. (**A**) Approximately 5×10^4^ cells were seeded on 2-chambered slides and starved in serum-free basal medium without or with fidarestat for overnight. The cells were washed with 1× HBSS and incubated with 10 µM H_2_DCF-DA at 37°C for 30 min, washed again and treated with IL-13 (25 ng/ml) for 1 h. The cells were washed with cold 1× HBSS twice and mounted using floursave mounting medium with DAPI. Photomicrographs were acquired using a fluorescence microscope (Nikon). A representative image is shown (n = 4); Magnification 400×. (**B**) Approximately 10,000 SAEC were plated per well in a 96-well plate and serum-starved for 24 h without or with fidarestat. The cells were washed with 1× HBSS and incubated with 10 µM H_2_DCF-DA at 37°C for 30 min. Cells were washed again to remove excess H_2_DCF-DA and treated with Il-13 (25 ng/ml) in basal media for 1 h. At the end of the treatment, cells were washed twice with HBSS and fluorescence was determined at 485 nm excitation and 538 nm emission wavelengths. Relative ROS production is expressed as mean fluorescence intensity (MFI) (arbitrary units). The bars represent mean ± SD (n = 4–6); (^*^ *p*<0.01 vs. Control; ^**^ *p*<0.05 vs. IL-13).

Since cytokines- induced oxidative stress is known to mediate molecular signaling that leads to differentiation of airway epithelial cells into mucus cells, so we determined the effects of AR inhibition on IL-13-induced ROS levels in SAEC by two different methods. As shown in Fig. 2A and 2B, stimulation with IL-13 caused approximately two-fold increase in the ROS levels over the control and treatment of the cells with fidarestat significantly (80%) prevented the increase. These results suggest that cytokine-induced oxidative stress could be prevented by inhibition of AR.

### AR inhibition prevents IL-13-induced goblet cell metaplasia in SAEC

**Figure 3:**
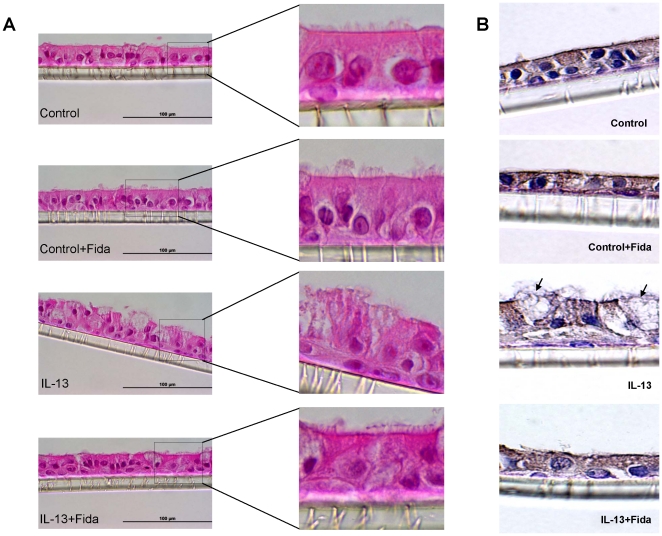
AR inhibition prevents IL-13-induced loss of cilia and β-tubulin in airway epithelial cell monolayer. Approximately 8.5×10^4^ SAEC were seeded per 12 mm diameter PET transparent insert and cultured for 7 days in differentiation media supplied from both lower and upper sides followed by culture on ALI for 11 days for differentiation into ciliated cells. The monolayer was treated with AR inhibitor for overnight and stimulated with IL-13 for 48 h. The monolayers on the membrane inserts were fixed for 24 h in z-fix (10% buffered formalin with zinc) at 4°C, paraffin embedded and 5 µM sections were cut. (**A**) The H&E stained sections were examined under the light microscope; magnification 400×. Inset shows magnified view of the selected regions from each representative photomicrograph (n = 4). (**B**) Immunohistochemistry using β-tubulin antibodies was performed on the sections. Dark brown staining in the monolayer corresponds to cilia axoneme β-tubulin. Arrows show loss of β-tubulin as the ciliated cells transform into goblet cells. A representative photomicrograph has been shown (n = 4); magnification 400×.

To investigate the role of AR in the regulation of goblet cells metaplasia and mucin production, we used an air-liquid interface culture system that mimics in-vivo airway environment. First, we cultured primary human airway epithelial cells on polyethylene terephthalate (PET) membrane insert with collagen type-1 coating. The cells on the collagen-coated membrane insert formed a consistent 2–3 cells thick layer of airway cells. Further, the monolayer cells fully differentiated into ciliated cells when cultured on the ALI in EGF-containing medium for 11 days (Fig. 3A; control) and when these cells were stimulated with IL-13 for 48 h, the number of ciliated cells were markedly reduced as also determined by immunostaining using β-tubulin specific antibodies. However, AR inhibition by fidarestat preserved the ciliated morphology (Fig. 3B). AR inhibitor alone had no toxic effects and the monolayers of ciliated cells were largely intact. As determined by PAS staining a large number of ciliated cells (∼6-fold) transformed into mucus-laden goblet cells when stimulated with IL-13 for 48 h, whereas addition of AR inhibitor, fidarestat, in the medium prior to IL-13 challenge prevented the airway cells from differentiating into goblet cells (data not shown).

### AR inhibition prevents IL-13-induced expression of Mucin and SPDEF in airway epithelial monolayer

**Figure 4:**
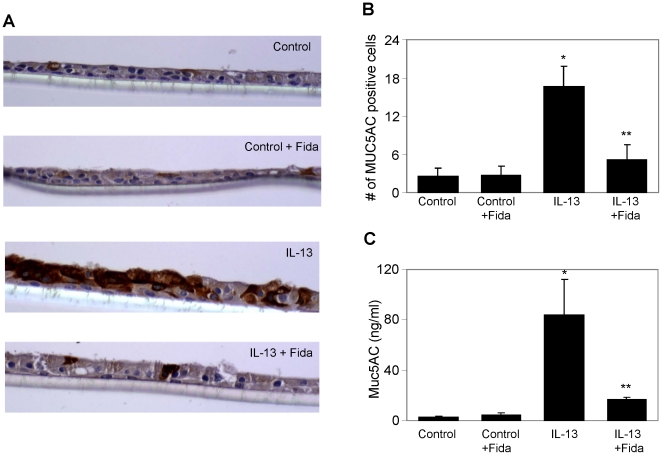
AR inhibition prevents IL-13-induced goblet cell metaplasia and expression of Muc5AC in airway epithelial monolayer. (**A**) The well differentiated airway epithelial cells at ALI were incubated with IL-13 for 48 h without or with AR inhibitor, fidarestat. The monolayer was fixed as described and immunohistochemistry using Muc5AC antibodies was performed on the sections. A representative photomicrograph has been shown (n = 4); magnification 400×. (**B**) The bar diagram shows number of Muc5AC positive cells per viewing area from 10 areas counted randomly from each section under the microscope (n = 4); *p<0.0005 vs Control; **p<0.004 vs IL-13. (**C**) The airway epithelial monolayer was incubated with IL-13 submerged in basal medium for 48 h in the absence and presence of AR inhibitor, fidarestat. The culture medium was collected from the top chamber and utilized for determination of Muc5AC by an ELISA, (n = 4); *p<0.005 vs Control; **p<0.01 vs IL-13.

**Figure 5:**
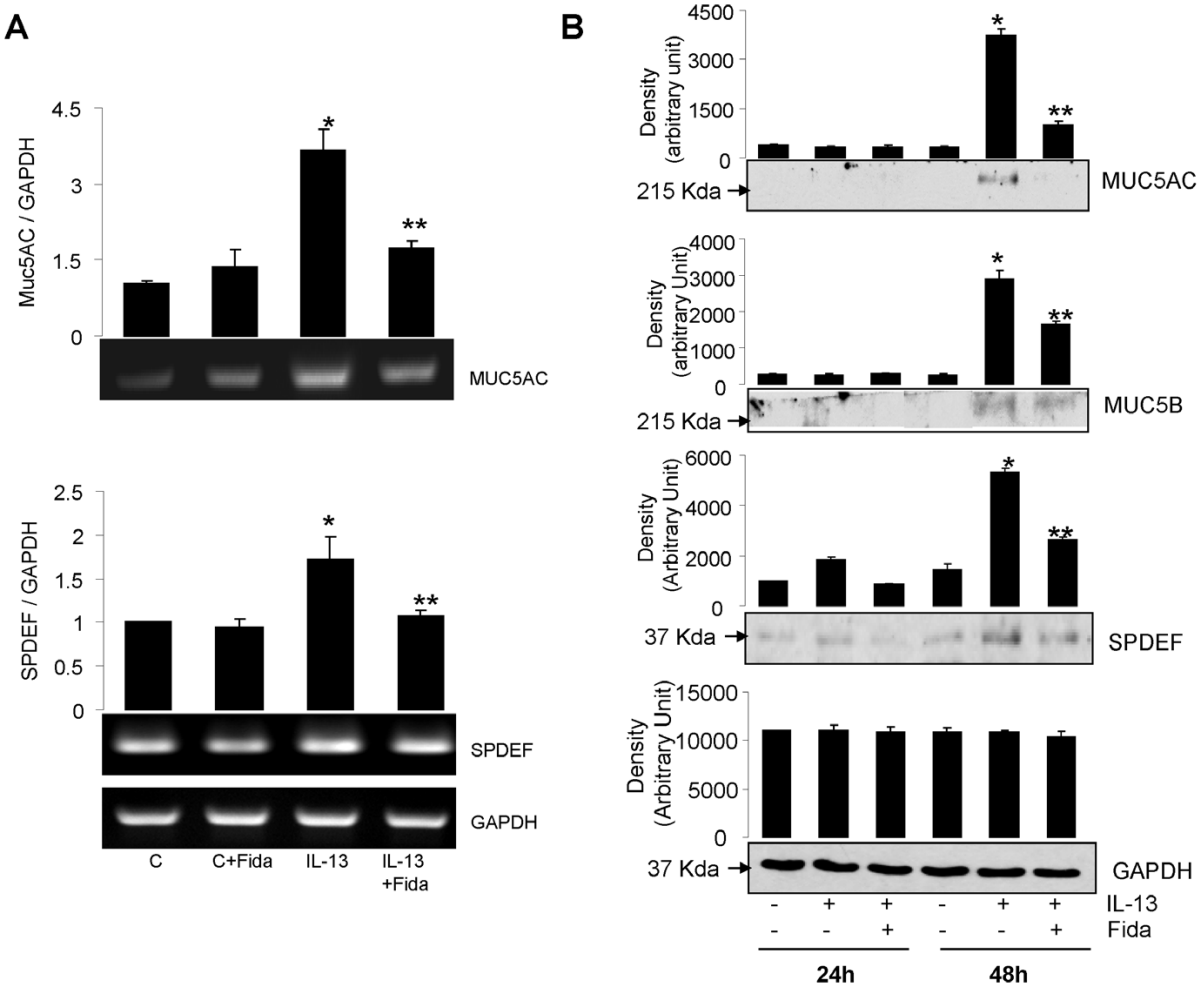
AR inhibition prevents IL-13-induced expression of Mucin and SPDEF in airway epithelial cell monolayer. (**A**) The airway epithelial cell monolayer at ALI was serum starved without or with fidarestat and incubated with IL-13 for 18 h. Total RNA was isolated and subsequently RT-PCR was performed to assess the expression of Muc5AC and SPDEF. The bar diagrams show densitometric analysis of the corresponding blots (n = 4). *p<0.001 vs Control; **p<0.001 vs IL-13; (**B**) The monolayer of ciliated airway cells at ALI was treated with AR inhibitor for 24 h and subsequently incubated with IL-13 for 24 or 48 h. At the end of incubation, cell lysate was prepared and subjected to western blotting using antibodies against Muc5AC, Muc5B, and SPDEF. The membranes were stripped and re-probed with antibodies against GAPDH to show the equal loading of protein. A representative blot is shown (n = 4). *p<0.0001 vs Control; **p<0.001 vs IL-13.

Next, we stained sections of monolayer cells with antibodies against Muc5AC. As shown in Fig. 4A and 4B, IL-13 stimulation resulted in a large number of Muc5AC immuno-positive cells. When the cells were treated with AR inhibitor and incubated with IL-13, the number of cells with Muc5AC positive staining decreased significantly (∼70%) suggesting that only a few cells had transformed into goblet cells. Next, we measured the level of secreted Muc5AC in the culture medium using an ELISA. There was a marked increase in the secretion of Muc5AC in the medium after IL-13 stimulation which was significantly (∼80%) prevented by treatment with AR inhibitor (Fig. 4C). These results suggest that AR regulates IL-13-mediated metaplasia and mucus production in the airway epithelial cells and inhibition of AR could prevent these events. Next, we examined whether Mucin levels in these cells were regulated by transcription or translation. As shown in Fig. 5A, upper panel, Muc5AC-specific RNA expression increased by approximately 3-fold in IL-13-treated SAEC monolayer and fidarestat significantly prevented the increase.

A number of studies have shown that SPDEF regulates the expression of several genes in the airway epithelial cells including both acidic and neutral mucins and cause goblet cell hyperplasia [20], [21]. We therefore, determined the expression of SPDEF mRNA levels by RT-PCR in our cell-culture model and observed that IL-13 significantly enhanced the expression of SPDEF in epithelial cells, whereas control cells had only basal level of expression. When these cells were treated with AR inhibitor prior to IL-13 stimulus, the SPDEF mRNA levels decreased significantly (Fig. 5A, lower panel).

Next we measured the levels of Muc5AC and Muc5B proteins by immunoblotting in SAEC monolayer after incubation with IL-13 for 24 and 48 h. Muc5AC/B were not detectable after 24 h however, after 48 h of incubation with IL-13 there was a robust increase in the expression of protein levels (Fig. 5B, top two panels), which was significantly (∼75 and ∼50%, respectively) prevented in fidarestat treated cells. Also in the airway epithelial cell monolayer incubated with IL-13, the levels of SPDEF protein in the cells significantly increased after 24 h and further increased after 48 h and the increase was significantly (∼50%) prevented by AR inhibitor treated cells (Fig. 5B). The decrease in the levels of protein corresponded with the decrease in mRNA levels of SPDEF and Muc5AC/B suggesting that SPDEF and Muc5AC genes are induced in the presence of IL-13, and that the expression of Muc5AC is prevented by AR inhibition at the level of transcription.

### AR inhibition prevents IL-13-induced phosphorylation and activation of signaling intermediates

**Figure 6:**
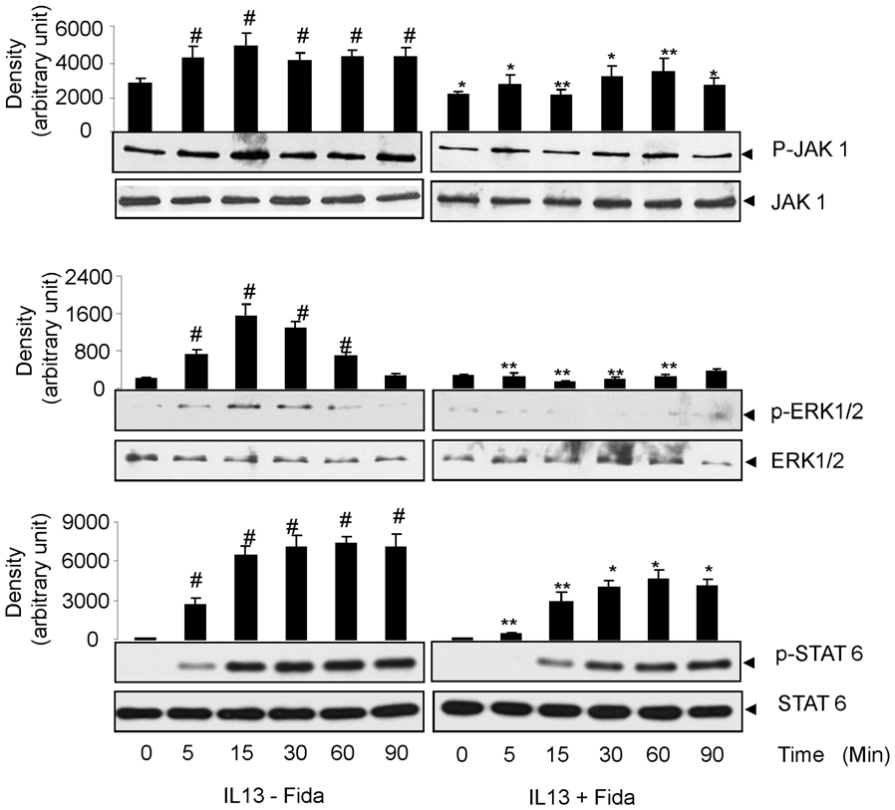
AR inhibition prevents IL-13-induced phosphorylation of JAK-1, ERK1/2 and STAT-6 in airway epithelial monolayer. The ciliated monolayer of airway cells at ALI was treated with AR inhibitor for 24 h and stimulated with IL-13 for different time periods as indicated. At the end of incubation, cells were lysed and cell lysate was subjected to western blotting using antibodies against phosphorylated and non-phosphorylated JAK-1, ERK1/2 and STAT-6 to analyze the activation of these signaling proteins. A representative blot is shown (n = 4). #p<0.001 vs Control; *p<0.01 and **p<0.001 vs IL-13.

Since AR inhibition in airway epithelial cells monolayer on ALI successfully prevented the expression of transcription regulator SPDEF and subsequent expression of mucin, we next investigated the effect of AR inhibition on the molecular mechanism(s) that regulates the expression of these mediators of asthma. We stimulated the cells grown on ALI with IL-13 in presence or absence AR inhibitor and determined the phosphorylation of JAK1, ERK1/2 and STAT-6 proteins. As shown in Fig. 6, IL-13 caused a time-dependent increase in the phosphorylation of STAT-6 and upstream mediators such as JAK1 and ERK1/2. When these cells were treated with AR inhibitor prior to IL-13 stimulus, the phosphorylation of these proteins decreased significantly. Further, in cells treated with AR inhibitor alone the basal phosphorylation of JAK1 but not ERK1/2 and STAT-6 was decreased. These results suggest that AR regulates the activation of key signaling intermediates involved in goblet cell metaplasia which transforms airway-ciliated epithelial cells to mucus secreting goblet cells.

### AR inhibition prevents RWE-induced expression of IL-13 and activation of STAT-6 in mouse lung

**Figure 7:**
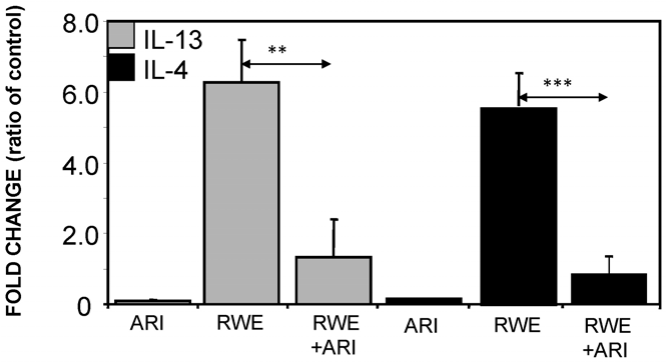
AR inhibition prevents RWE-induced expression of IL-13 in mouse lung. The mice were sensitized and challenged with PBS or RWE, without or with AR inhibitor and 16 h later lungs were harvested and total RNA was isolated (n = 4). One microgram of total RNA from each sample was transcribed into first-strand cDNA and quantitative RT-PCR was conducted using IL-13 specific forward and reverse primers. The levels of RNA for the target sequences were determined by melting curve analysis. The values presented here are fold-change over the control. (^**^ *p*<0.001). RWE, ragweed pollen extract; ARI, aldose reductase inhibitor.

**Figure 8:**
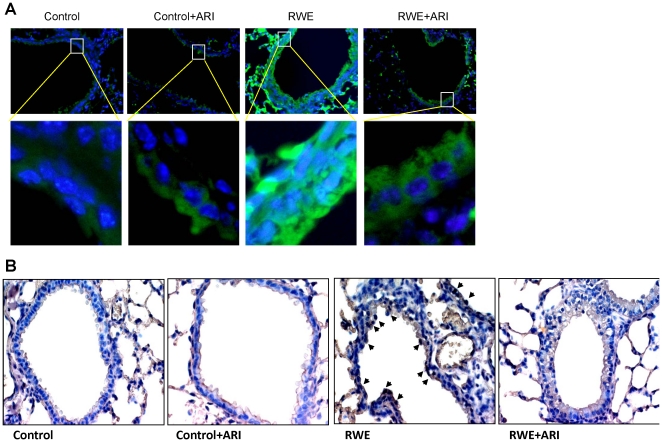
AR inhibition prevents phosphorylation of STAT-6 in mouse lung epithelium. The mice were sensitized and challenged with PBS or RWE, without or with AR inhibitor and 20 h later lungs were perfused and fixed with 4% paraformaldehyde, embedded in paraffin, and sectioned to 5 µm. The sections were immunostained with p-STAT-6 specific antibodies using immunoflouroscence secondary antibodies (**A**) or DAB –based HRP conjugated antibodies counterstained with hematoxylin and eosin (**B**). Photomicrographs were acquired by fluorescence or light microscopes. A representative field for each group is shown (magnification: 200×). In (A) inset shows magnified view of the selected regions from representative photomicrographs (n = 4). RWE, ragweed pollen extract; ARI, aldose reductase inhibitor.

Since Th2 cytokines, especially IL-13, is involved in the goblet cell metaplasia, we examined the expression levels of IL-13 in mice lung after RWE challenge and found that its level increased approximately 6-fold compared to control and treatment with fidarestat prior to challenge prevented this increase (Fig. 7). To examine whether increased IL-13 levels coincided with the phosphorylation and activation of STAT-6 which play a major role in metaplasia, we killed the mice after 20 h of RWE challenge and performed immunofluorescence studies on the lung section using phospho-STAT-6 antibodies. We observed that while the lungs of control mice showed only background, RWE-challenged mice lung epithelium showed a marked increased in the fluorescence intensity specific to phospho-STAT-6, which was prevented by AR inhibition (Fig. 8A). We confirmed our results by DAB-based immunohistochemistry on the lung sections as well (Fig. 8B). These results suggest that inhibition of AR could prevent the allergen-induced IL-13 expression and subsequent activation of STAT-6 in vivo.

### AR inhibition/deficiency prevents RWE-induced goblet cell metaplasia in mice lung

**Figure 9:**
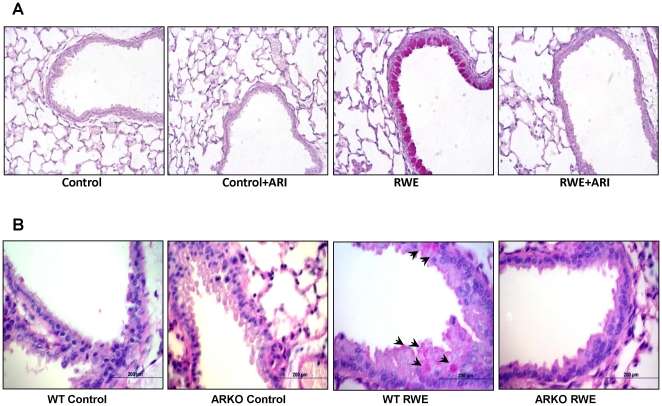
Inhibition or deficiency of AR prevents RWE-induced goblet cell metaplasia in mice lungs. RWE-sensitized normal and AR-null mice were challenged with RWE and 72 h later the lungs were harvested from mice treated without or with AR inhibitor (**A**) or AR-null mice (**B**), perfused and fixed with 4% paraformaldehyde and embedded in paraffin. The sections were stained with PAS stain and observed under light microscope and photomicrographs were acquired. A representative photomicrograph from each group is shown (n = 4). Magnification 200× (A); 400× (B).

We next examined the effect of AR inhibition on goblet cell metaplasia in RWE-sensitized and-challenged mice. After 72 h of challenge the lung sections were obtained and stained with PAS and changes in the airway epithelia were examined. As shown in Fig. 9A, there was a significant increase in the PAS positive cells in the airway of RWE-challenged mice and absent in the lungs of mice treated with fidarestat prior to RWE challenge. Similar to the inhibitor-treated mice, AR-null mice challenged with RWE showed absence of PAS positive cell in the airway (Fig. 9B). These results further confirm that AR plays a significant role in the goblet cell metaplasia and AR inhibition could prevent metaplasia in allergic asthma.

## Discussion

Allergens are known to activate macrophages and Th2 lymphocytes which generate various cytokines and chemokines that activate NADPH oxidase and generate excessive amounts of ROS, a major contributor to allergic asthma [36]–[41]. We have demonstrated earlier that ROS causes lipid peroxidation and the reduced lipid-aldehyde-glutathione conjugates activate various kinases which through the activation of transcription factors induce expression of pro-inflammatory chemokines/cytokines that increase ROS levels in autocrine and paracrine manner and exacerbate asthma pathogenesis [28]–[31]. In addition, ROS activate JAK-STAT pathway which in turn contributes to goblet cell metaplasia, a major player in mucin production and airway clogging [19], [23], [42], [43]. Recently, Severgnini et al showed that ROS regulates STAT activation in acute lung injury [44]. Similarly, Simon et al demonstrated that PDGF-dependent activation of STAT in airway smooth muscle cells is redox-dependent [45]. Further, treatment of antioxidants such as N-acetyl-L-cysteine (NAC) prevented the activation of JAK-STAT pathway indicating that the ROS plays a crucial role in this pathway [43], [45]. Activation of JAK-STAT pathway is involved in the allergen-induced eosiniphilia, airway hyperresponsiveness and mucus hypersecretion [19], [23], [24]. Indeed, we have demonstrated that SAEC exposed to RWE or TNF-α generate excessive amounts of ROS [26], [46]. To further elucidate the role AR and potential clinical utility of its inhibitor fidarestat, we examined goblet cells metaplasia in exposed SAEC to IL-13, a Th2 cytokine and mouse model of asthma.

We cultured SAEC ± IL-13 and quantified ROS. A significant (∼2-fold) increase in the ROS levels in SAEC exposed to IL-13 and prevention by AR inhibition (Fig. 2) suggests that AR inhibition could play an anti-oxidative role. To investigate the effects of AR inhibition on the goblet cell metaplasia, we cultured SAEC on ALI, where they formed an epithelial monolayer with cilia on the apical surface. Stimulation with IL-13 transformed majority of the cells into mucus-filled goblet cells which lacked cilia. Further, when the airway cells in monolayer were incubated with AR inhibitor prior to IL-13 stimulation, metaplasia was prevented as the number of cells with PAS staining and Muc5AC immuno-reactivity significantly decreased and the cells in monolayer maintained ciliated morphology. It has also been shown that Th2 cytokines-induced increase in oxidative stress increased number of mucus producing goblet cells in airway epithelium, which secrete excessive amounts of mucus, especially Muc5AC [6]–[10]. Furthermore, activation of JAK-STAT and MAPK pathways by allergen-induced increase in IL-13 in allergic asthma has been demonstrated in goblet cell metaplasia [25], [47]. Our results that AR inhibition significantly prevented IL-13-induced phosphorylation of JAK-1, ERK1/2 and STAT-6 in airway epithelial cells suggest that AR could regulate the redox signaling that is known to cause goblet cell metaplasia. SPDEF orchestrate a transcriptional program for goblet cells differentiation, and has been implicated in goblet cell metaplasia in response to allergen or IL-13 challenge [20], [21]. SPDEF transgenic mice showed increased where as deletion of SPDEF gene in mice prevented goblet cell formation in the conducting airway epithelium subsequent to pulmonary allergen exposure [21], [23]. Our results suggest that prevention of IL-13-induced expression of SPDEF and Muc5AC in airway epithelial cells by AR inhibition may contribute to decreased metaplasia in AR inhibitor-treated cells (Fig. 5). These observations thus indicate that AR inhibition could alter the redox status of cells and prevent the activation of JAK-1, ERK1/2 and STAT-6 and decrease the expression of mucin genes and their regulators thereby prevent goblet cell metaplasia.

Prevention of goblet cell metaplasia by AR inhibition observed in cultured airway epithelial cells was further confirmed by using a mice model of RWE-induced allergic asthma. The population of PAS positive goblet cells in the RWE-challenged mice lung was significantly less in the mice treated with fidarestat. Further, to rule out any nonspecific effect of the inhibitor, we used AR-knock out mice in which RWE did not cause goblet cell metaplasia as observed by the absence of PAS-stained goblet cells in airway epithelium. These results further substantiate that AR inhibition or deficiency could prevent the allergen-induced goblet cell metaplasia. In order to further understand how AR inhibition prevents allergen-induced goblet cell metaplasia, we examined the effect of AR inhibition on the levels of IL-13 expression and STAT-6 phosphorylation in the mice lungs. In the RWE-challenged and fidarestat-treated mice there was significantly (80%) decreased IL-13 expression compared to RWE-challenge without AR inhibitor. The decrease in IL-13 correlated with decrease in STAT-6 phosphorylation and prevention of metaplasia by AR inhibition.

We have demonstrated it earlier that AR-catalyzed reduced glutathione-lipid aldehydes such as GS-DHN via activation of various protein kinases activate transcription factors which transcribe inflammatory marker genes and inhibition of AR not only prevents the transcription of inflammatory markers, it also significantly blocks ROS generation thereby prevent further amplification of this cycle [31], [32]; Fig. 1). Although antioxidants such as n-acetylcysteine (NAC), genistein and curcumin, caffeic acid, alpha-lipoic acid, vitamin C and vitamin E have been suggested earlier to attenuate airway inflammation in animal models of asthma [48]–[55], none have been successfully developed for clinical use in human. Since inflammatory cytokines and subsequent generation of ROS are major contributors to asthma pathogenesis including mucus hypersecretion, our results indicate that AR inhibitors, such as fidarestat, could be developed to prevent goblet cell metaplasia and mucus hypersecretion since this inhibitor has already gone through phase III clinical trial for diabetic neuropathy and found to be safe.

## Materials and Methods

### Ethics statement

All animal experiments were performed according to the National Institutes of Health Guide for Care and Use of Experimental Animals and approved by University of Texas Medical Branch Animal Care and Use Committee (Animal welfare assurance No. A3314-01).

### Reagents

Small airway epithelial basal medium (SABM), and small airway epithelial growth media (SAGM™) bulletkit; and Reagentpack™ containing Trypsin 0.025%/EDTA 0.01%, Trypsin neutralizing solution and HEPES buffered-saline solution were purchased from Lonza Walkersvillle Inc. (Walkersville, MD). Dulbecco's modified Eagle's medium (DMEM) and phosphate buffered saline (PBS) were purchased from Gibco, Invitrogen (Grand Island, NY). AR inhibitor, fidarestat, was a gift from Sanwa-Kayagu (South Korea). Human recombinant IL-13 was from R & D systems (Minneapolis, MN). Dimethyl sulfoxide (DMSO) was obtained from Fischer scientific (Pittsburg, PA). Human mucin5AC ELISA kit was from Cosmo Bio USA (Carlsbad, CA). Antibodies against STAT- 6, phospho-STAT- 6, phospho-JAK1, JAK1, ERK1/2, phospho-ERK1/2 were from Cell Signaling Tech. (Danvers, MA) and mucin 5 subtypes A and C (Muc5AC), Muc5B, GAPDH and β-actin antibodies were from Santa Cruz Biotechnology Inc. (Santa Cruz, CA). Antibodies against SAM pointed domain-containing ETS transcription factor (SPDEF) were purchaged from Abcam Inc. (Cambridge MA). The polyethylene pteraphthalate transparent 12-well Millicell cell culture insert with 1.0 µM pores were purchased from Millipore Corp. (Billerica, MA). Rat tail collagen type 1 and all trans-retinoic acid were from Sigma-Aldrich (Saint Louis, MO). The reagents used in the Western blot analysis were obtained from Sigma. All other reagents used were of analytical grade.

### Cell culture

Primary human Small Airway Epithelial Cells (SAEC) obtained from Lonza Walkersville, Inc. (Walkersville, MD) were normal human SAEC harvested from distal airspace. The cells were cultured and maintained according to the supplier's instructions at 37°C in humidified atmosphere containing 95% air and 5% CO_2_ in small airway epithelial basal medium (SABM) supplemented with 52 µg/ml bovine pituitary extract, 0.5 ng/ml human recombinant epidermal growth factor (EGF), 0.5 µg/ml epinephrine, 1 µg/ml hydrocortisone, 10 µg/ml transferrin, 5 µg/ml insulin, 0.1 ng/ml retinoic acid (RA), 6.5 ng/ml triiodothyronine, 50 µg/ml Gentamicin/Amphotericin-B (GA-1000), and 50 µg/ml fatty acid-free bovine serum albumin (BSA).

### ROS levels determination

Approximately 5×10^4^ SAEC were seeded on 2-chambered culture slides in triplicate or 10,000 SAEC per well were plated in a 96-well plate. After they attached, cells were starved in basal medium containing 0.1% serum without or with fidarestat (10 µM) for overnight. Next day cells were washed with 1× HBSS buffer and incubated with 10 µM H_2_DCF-DA at 37°C for 30 min, washed again to remove excess H_2_DCF-DA and treated with IL-13 (25 ng/ml) for 1 h. At the end of incubation, cells were washed twice with cold 1× HBSS buffer. The cells on the culture slide were mounted using floursave mounting medium with DAPI after which photomicrographs were acquired using a fluorescence microscope (Nikon). Fluorescence was determined using 485 nm excitation and 538 nm emission wavelengths for cells in 96-well plate and relative ROS production is expressed as mean fluorescence intensity (MFI) (arbitrary units).

### Air-liquid interface culture

For air-liquid interface (ALI) culture, SAEC were seeded at 8.5×10^4^ cells/12-mm-diameter PET transparent insert with 1.0 µM pores (12-well millicell culture insert; Millipore) and pre-coated with rat tail collagen type-1 (Sigma-Aldrich). The cells were grown submerged in the differentiation medium as described earlier by Zhen et al (2007) [56]. The differentiation medium contained a 1∶1 mixture of DMEM and small airway epithelial growth medium supplemented as described above, except that gentamycin sulfate, amphotericin B, and triiodothyronine were replaced with 1% penicillin/streptomycin, and 50 nM all-trans retinoic acid. The SAEC were maintained submerged for the first 7 days, after which the apical medium was removed and an air–liquid interface culture was established. The cells were maintained at ALI for the remainder of the culture period. Medium was refreshed every third day and once in a week the apical surface of the cells was rinsed with PBS to remove accumulated mucus and debris. Cells were maintained at 37°C in 95% air and 5% CO_2_ in a humidified incubator. The cells on the ALI were pre-treated with AR inhibitor, fidarestat (10 µM), for over-night from the basal side in differentiation medium without EGF beginning day 11 after establishment of ALI and stimulated with IL-13 by addition of recombinant human IL-13 (25 ng/ml) to the medium for varying time periods as indicated.

### Cell fixation and immunocytochemistry

After the completion of incubation with IL-13, the apical surface of the cells was rinsed with PBS and cells were fixed in 10% z-fix, aqueous buffered-zinc formalin (Anatech Ltd; Battle Creek, MI), for 24 h at 4°C and embedded in paraffin. The 5 µM thin sections of the epithelial cell monolayer on the membrane insert were stained with H&E and periodic acid Schiff (PAS)-stain. The stained sections were analyzed and representative fields were photographed using a Photometrix CoolSNAP Fx camera mounted on a NIKON Eclipse TE 200 UV microscope.

Antibodies against β-tubulin and Muc5AC (Santa Cruz Biotechnology, Santa Cruz, CA) and matched control IgG were used for immunocytochemistry. Antibodies were detected using the Vector LSAB kit (Vector Laboratories, Burlingame, CA) as suggested by the manufacturer.

### RT-PCR

Total RNA was isolated from differentiated SAEC treated with IL-13 with or without AR inhibitor by using RNeasy kit (Qiagen) as per supplier's instructions. Aliquots of RNA (1.0 µg) isolated from each sample were reverse-transcribed with Omniscript and Sensiscript reverse transcriptase one-step RT-PCR system with HotStar Taq DNApolymerase (Qiagen) at 55°C for 30 min followed by PCR amplification. The oligonucleotide primer sequences were as follows: Muc5AC: 5′-TCCGGCCTCATCTTCTCC-3′ (sense) and 5′-ACTTGGGCACTGGTGCTG-3′ (Antisense); SPDEF: 5′-CGAAGTGCTCAAGGACATCGAG-3′ (sense) and 5′-CGGTATTGGTGCTCTGTCCACA-3′ (anti-sense) and GAPDH: 5′-GACCCCTTCATTGACCTCAAC-3′ (sense) and 5′-CATACCAGGAAATGAGCTTG- 3′ (antisense). RT-PCR reaction was carried out in a PCR Sprint thermal cycler (Thermo electron corporation, Milford, MA) under the following conditions: initial denaturation at 95°C for 15 min followed by 35 cycles at 94°C for 1 min, 60°C for 1 min, 72°C for 1 min, followed by 72°C for 10 min for final extension. The RT-PCR products were subjected to electrophoresis on a 1.5% agarose-1X TAE gels containing 0.5 µg/ml ethidium bromide. The densitometry analysis of the gel was performed using NIH image analysis software.

### Western blot analysis

Subsequent to incubations, the cells were washed with cold PBS and lysed in RIPA lysis buffer. The cell lysates were pooled and cleared by centrifugation. Protein levels were determined using Bradford reagent (Biorad, Hercules, CA). Forty micrograms of protein were mixed with sample buffer and resolved on 10% SDS-PAGE. After electrophoresis, the proteins were electro transferred to a nitrocellulose membrane, blocked with 5% nonfat milk in TBST, and probed with antibodies against phospho-ERK1/2, ERK1/2, phospho-STAT-6, STAT-6, Muc5AC, Muc5B, and SPDEF for overnight at 4°C. The blots were then washed, exposed to HRP-conjugated secondary antibodies (1∶5,000 dilution) for 1 h, and the antigen-antibody complex was detected by enhanced chemiluminescence (Amersham Pharmacia Biotech, Piscataway, NJ, USA). The membranes were stripped and reprobed with antibodies against GAPDH to depict loading control. Densitometry was performed by biospectrum 410 image system from Ultra Violate Products Ltd. (Cambridge, UK).

### Muc5AC ELISA

Muc5AC levels in the culture medium were assessed by ELISA using commercially available human anti-Muc5AC ELISA essentially as described by the manufacturer (Cosmo Bio USA; Carlsbad, CA).

### Sensitization and challenge of animals

Wild type C57BL/6 and Balb/cJ mice were purchased from Harlan Sprague-Dawley (San Diego, CA, USA) and AR null mice on C57BL/6 background were bread by us at Animal resource center, UTMB, Galveston, TX. Six-eight weeks old female mice were sensitized with RWE as previously described [35]. Briefly, mice were sensitized with two intraperitoneal administrations of 100 µl of endotoxin-free RWE (150 µg) combined with Alum adjuvant (1 mg) in a 3∶1 ratio (v/v), on days 0 and 4. On day 11, mice (*n* = 6) were challenged intranasally with RWE (100 µg). A parallel group of mice received fidarestat (7 mg/kg body wt/day) in drinking water from one day before the challenge. Control groups of mice were challenged with equivalent volumes of PBS. The animals were euthanized at different time points as mentioned, with ketamine (135 mg/kg body wt) and xylazine (15 mg/kg body wt), the lungs were perfused and fixed with 4% paraformaldehyde, embedded in paraffin, and sectioned to 5 µm. Lung sections were stained with PAS and the representative fields were observed and photographed with a Photometrix CoolSNAP Fx camera mounted on a NIKON Eclipse TE 200 UV microscope.

### Determination of IL-13 in mice lungs

The mice lungs were harvested 16 h after RWE-challenge and total RNA was isolated. From each sample, one microgram of total RNA was transcribed into first-strand cDNA and quantitative RT-PCR was conducted using IL-13 specific forward and reverse primers 5′AGACCAGACTCCCCTGTGCA, 3′TGGGTCCTGTAGATGGCATTG; GAPDH specific primers (5′TGTGTCCGTCGTGGATCTGA, 3′CCTGCTTCACCACCTTCTTGAT) were used as house keeping gene (HKG) control. Data were collected and analyzed by ABI 7000 System equipment and software (Applied Biosystems, Foster City, CA). To assess expression levels, delta-delta Ct method (ΔΔC_T_) was used. Values of expression in fold increase (ratio of control) were calculated using the formula for relative expression by the method of Delta DeltaC_T_ (ΔΔC_T_): F = 2^−ΔΔC^. F = fold change (ratio of control), −ΔΔC_T_ =  (C_T_ Target-CT_HKG_)^Time *x*^ − (C_T_ Target-C_T HKG_)^Time 0^. Time *x* is any time point. Time 0 represents 1× expression of the target gene normalized to a HGK.

### Detection of STAT-6 phosphorylation in mouse lungs

Approximately 20 h after RWE-challenge, mice were killed and lungs were perfused and fixed with 4% paraformaldehyde, embedded in paraffin, and 5 µm sections were obtained. The sections were immunostained with p-STAT-6 specific primary antibodies followed by probing with either FITC labeled secondary antibodies and mounted with frourosave medium with DAPI or DAB –based HRP-conjugated antibodies from Vector LSAB kit (Vector Laboratories, Burlingame, CA) and counterstained with hematoxylin and eosin. Photomicrographs were acquired by Photometrix CoolSNAP Fx camera mounted on a NIKON Eclipse TE 200 UV microscope using fluorescence or bright-field microscopy, respectively.

### Statistics

Data presented as mean ± SE and statistical significance was determined by unpaired Student's t test using graph pad prism software (GraphPad Software, Inc. La Jolla, CA). The value of *P*<0.05 was considered as statistically significant.
